# Fetuin-A in Metabolic syndrome: A systematic review and meta-analysis

**DOI:** 10.1371/journal.pone.0229776

**Published:** 2020-03-05

**Authors:** Xiongfeng Pan, Shi Wu Wen, Prince L. Bestman, Atipatsa C. Kaminga, Kwabena Acheampong, Aizhong Liu

**Affiliations:** 1 Department of Epidemiology and Health Statistics, Xiangya School of Public Health, Central South University, Changsha, China; 2 Department of Obstetrics and Gynaecology, University of Ottawa, Ottawa, Ontario, Canada; 3 Ottawa Hospital Research Institute, Ottawa, Ontario, Canada; 4 Association of Liberian Anaesthetist, Monrovia, Liberia; 5 Department of Mathematics and Statistics, Mzuzu University, Mzuzu, Malawi; 6 Department of Public, School of Postgraduate Studies, Adventist University of Africa, Nairobi, Kenya; Karolinska Institutet, SWEDEN

## Abstract

**Objective:**

Fetuin-A has been associated with the progression of metabolic syndrome, but previous studies found inconsistent results on the relationship between metabolic syndrome and the concentration of fetuin-A. The aim of this study was to perform a meta-analysis to summarize previous findings on this relationship.

**Method:**

This study was registered with the International Prospective Register of Systematic Reviews PROSPERO (CRD42019129566). Studies examining the relationship between metabolic syndrome and the concentration of circulating fetuin-A were identified using a systematic search in the electronic databases of Embase, PubMed, Web of Science, and Cochrane Library before March 2019. A random effects model was used to summarize the effect size of the association in terms of the standardized mean difference (SMD).

**Results:**

Fourteen eligible studies compared fetuin-A concentrations between 4,551 metabolic syndrome patients and 8,805 controls. The circulating fetuin-A concentration was significantly higher in the metabolic syndrome patients than in the controls (SMD = 0.65, 95% confidence interval (CI): 0.48 to 0.83, Z = 7.18, *p<*0.001). Besides, circulating fetuin-A was a risk factor for metabolic syndrome (odds ratio 1.23, 95% CI: 1.08 to 1.40).

**Conclusion:**

These findings suggest that fetuin-A may be an important indicator for metabolic syndrome, in which case this may lead to new perspectives in early diagnosis, identification of novel biomarkers, and providing novel targets for pharmacological interventions.

## 1. Introduction

Metabolic syndrome (MetS) is a new epidemic worldwide and it is associated with an increased risk for developing other chronic disease such as diabetes mellitus [1]. The cardinal features of MetS include the following medical conditions: central obesity, hypertension, glucose intolerance, hyper-triglyceridemia, and low serum high-density lipoprotein (HDL) [2]. It has been proposed recently that biomarkers such as circulating fetuin-A may have prognostic value for MetS [3]. Specifically, circulating fetuin-A is produced from adipose and hepatic tissue, secreted by paracrine, autocrine, and endocrine glands, also referred to as α2-Heremans-Schmid glyco-protein. Therefore, it has been widely recognized as a multifunctional molecule that participates in many metabolic processes, including energy expenditure, appetite control, insulin resistance, and regulation of adipogenesis [4].

**Fig 1:**
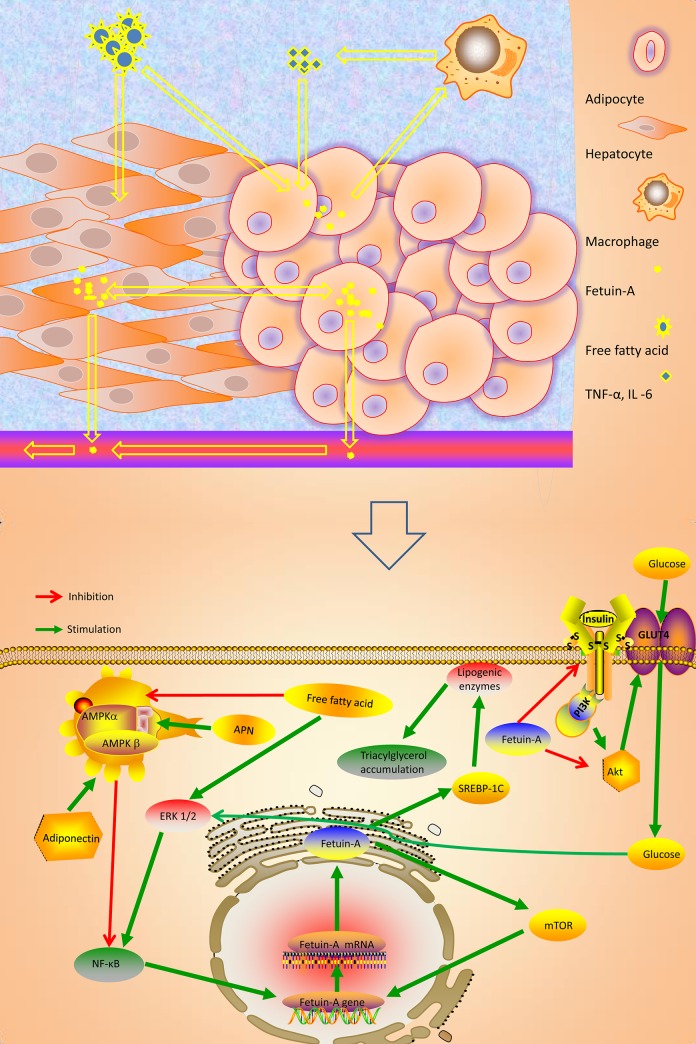
Summarizes the hypothesis mechanism process of fetuin-A on glucolipid metabolism signaling cascade in MetS. Fig 1 Schematic representation of the effects of fetuin-A on glucolipid metabolism signaling cascade in MetS that explain positive effects of these factors on glucolipid control. MetS, Metabolic syndrome; Akt: protein kinase b; IL -6: interleukin-6; TNF-α: Tumor Necrosis Factor; AMPK: AMP activated protein-kinase; GLUT: glucose transporter; IRS: insulin receptor substrate; mTOR: mammalian target of rapamicin; APN, adiponectin; PI3K: phosphatidylinositol 3-kinase; mTOR, mechanistic target of rapamycin; NF-κB, nuclear factor κB; ERK 1/2, Extracellular signal-regulated kinases 1 and 2; SREBP- 1C, sterol regulatory element binding protein-1c. Fig 1 is drawn by the KA, without any copyright disputes.

Several hypothetical mechanisms that play major roles in patients with MetS are being revealed. For example, it has been proposed that circulating fetuin-A modulates the insulin sensitivity and insulin resistance in organs such as adipose, liver, and other tissues [5]. According to existing data, a high calorie diet leads to free fatty acid excess and insulin resistance, hence fueling circulating fetuin-A synthesis and steatosis. Besides, circulating fetuin-A sends the chemo-attractant signals that induce macrophage secretion by hepatocytes and adipocytes, which is infiltrated into adipose tissue. Thus, the activated macrophage increases the expression of inflammatory cytokines, such as tumor necrosis factor (TNF-α) and interleukin-6 (IL-6), which contribute to subsequent steatosis and glucose metabolism disorder [6] (Fig 1). On the other hand, circulating fetuin-A inhibits the activity of insulin receptors and leads to insulin resistance. These effects are mediated through the phosphatidylinositide 3-kinase (PI3K) and Akt signaling pathways [7]. Meanwhile, circulating fetuin-A inhibits the activity of glucose transporter type 4 (GLUT4) translocation, and glucose uptake is impaired, as shown in Fig 1 (drawn by the KA).

Taken together, the preceding hypotheses suggest that circulating fetuin-A is a complex metabolic phase reactant with a debated role in MetS. However, studies investigating the association between circulating fetuin-A and MetS found inconsistent results [8, 9]. Therefore, this study aimed to conduct a comprehensive meta-analysis on the relationship between circulating fetuin-A and MetS, and to quantify the strength of this relationship. Moreover, this study conducted meta-regression to explore the potential influencing factors affecting this relationship.

## 2. Methods

### 2.1. Search strategy

This meta-analysis was conducted in accordance with the rules and regulations for meta-analysis in the Cochrane Handbook version 5.1.0. In addition, it was registered with the International Prospective Register of Systematic Reviews PROSPERO (CRD42019129566). The results were reported according to the Preferred Reporting Items for Systematic Reviews and Meta-Analyses (PRISMA) checklist [10]. With the help of experienced librarians, the researchers identified English articles, which were published before March 2019, from the electronic databases of Web of science, Cochrane library, Embase, and PubMed, using predefined search strategies. Detailed search strategies are described in the S1 File.

### 2.2. Eligibility criteria

Studies meeting the following criteria were included in this meta-analysis: (1) case-control studies; (2) studies which reported diagnostic criteria for MetS; (3) studies that provided mean and standard deviation (SD) or odds ratio (OR) of circulating fetuin-A, or these could be obtained from authors as required; (4) studies were peer-reviewed before publication; and (5) studies were published in English. On the other hand, studies meeting the following criteria were excluded: (1) reviews or case reports; (2) MetS was studied in combination with other diseases; (3) the circulating fetuin-A was pharmacologically challenged before circulating fetuin-A measurement; (4) studies of animals; and (5) grey literature (unpublished literature). After independently screening articles, two reviewers [KA and AK] selected eligible studies and submitted them to the third reviewer [AL] who made the final decision.

### 2.3. Data extraction

Using a standardized data extraction form, methodological and outcome variables of interest were collected from each study as follows: (1) the first author's last name and year of publication; (2) characteristics of subjects such as Body Mass Index (BMI), mean age (mean, SD), and gender; (3) region of study; (4) laboratory measures such as fasting plasma glucose (FPG), diastolic and systolic blood pressure, HDL, and triglycerides; (5) biological sample characteristics such as material of sample, and mean concentration of circulating fetuin-A (mean, SD) or OR associated with the concentration of circulating fetuin-A; and (6) assay methods and storage temperatures. Two reviewers [BP and AK] used EpiData 3.0 and Excel 2007 to organize and save the extracted data. Any discrepancies were resolved by consensus.

### 2.4. Quality evaluation

To assess the quality of the eligible studies for this meta-analysis, the Newcastle-Ottawa Scale (NOS) was used [11]. This is a nine-star rating system designed for non-randomized studies. The NOS contains three domains and eight items. The three domains consist of the following broad perspectives: (1) Selection; (2) Comparability; and (3) Outcome. According to the NOS criteria, the studies were rated low, moderate, and high quality in accordance with the scores, 0–3, 4–6 and 7–9, respectively.

### 2.5. Statistical analysis

The ‘meta’ and ‘metafor’ packages in R software (version R 3.4.3) were used to perform meta-analysis. Given the variation in the populations and criteria used to define outcomes, a random effects model was used to pool the estimates from the eligible studies [12]. Using this model, the 95% confidence intervals (CIs) and corresponding odds ratios (ORs) were merged to compare the impact of internal exposure as regards the risk of major outcomes. In addition, the standardized mean differences (SMDs) and corresponding 95% CIs were merged into a single standardized mean difference (SMD) and corresponding 95% confidence interval (CI), as Cohen’s d, which were used to evaluate the strength of the relationship between MetS and the concentration of circulating fetuin-A. The SMD was considered to be low if lower than 0.5, moderate if between 0.5 and 0.8, or high if greater than 0.8 [13]. For all the meta-analyses, the level of heterogeneity was assessed using Cochrane Q test and measured by *I*^*2*^ statistic. The heterogeneity was considered high when the *I*^*2*^ was greater than 75%, moderate when the *I*^*2*^ was 25%~75% and low when the *I*^*2*^ was less than 25% [14].

When heterogeneity was high, meta-regression analysis was conducted to explore the source of heterogeneity [15]. The following variables were considered for the meta-regression analysis: material (Plasma = 0, Serum = 1), region (Other = 0, Asia = 1), NOS (Other = 0, High = 1), gender (Female = 0, Male = 1), BMI (<30 = 0, ≥30 = 1), age (<18 = 0, ≥18 = 1), FPG (<100mg/dL = 0,≥100mg/dL = 1), systolic blood pressure (<130mmHg = 0,≥130mmHg = 1), diastolic blood pressure (<85mmHg = 0,≥85mmHg = 1), HDL (≥40mg/dL = 0, <40mg/dL = 1), and triglycerides (<150mg/dL = 0, ≥150mg/dL = 1). We used random-effect restricted cubic splines, with three knots at the 25%, 50% and 75% percentiles of the distribution, to examine a potential non-linear dose–response relationship between circulating fetuin-A concentrations and risk of MetS. Sensitivity analysis was performed to test whether exclusion of individual studies had a significant impact on the overall outcomes. Subgroup analyses were conducted to explore the impact of characteristics of patients on the outcomes. Finally, publication bias was assessed using the Egger funnel plot and Egger’s linear regression test, when the number of studies reporting meta-analysis results was 10 or more [16]. In all the statistical tests, *p* values were calculated as two-sided and considered significant if less than 0.05.

## 3. Results

### 3.1. Literature search

**Fig 2:**
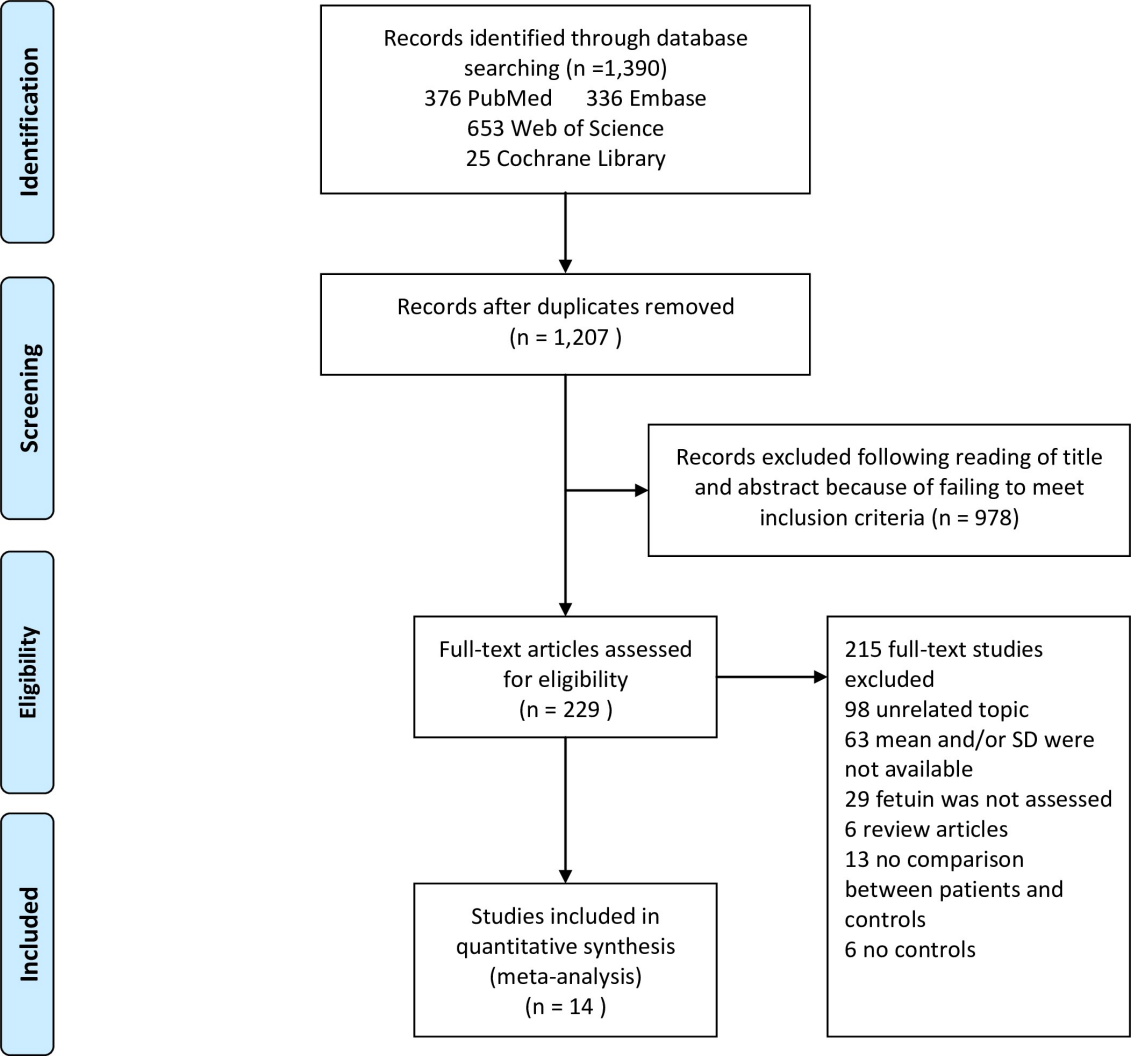
Flowchart of study selection. Showing the process by which relevant studies were retrieved from the databases, assessed, and selected, or excluded. Preferred reporting items for systematic reviews and meta-analyses (PRISMA) diagram for study search.

Fig 2 shows the selection process of the eligible articles for this meta-analysis. Initially, a total of 1,390 articles were identified from the four electronic databases as follows: 336 from Embase, 25 from the Cochrane Library, 653 from Web of Science and 376 from PubMed. After excluding the duplicates, 1,207 articles were retained. Also, after reviewing the titles and abstracts of the 1,207 articles, 978 articles were excluded because they did not meet the inclusion criteria. A full text review of 229 articles further excluded 98 for being unrelated studies, 29 for not reporting data on circulating fetuin-A, 63 for not reporting mean and SD of the concentration of circulating fetuin-A, 13 for not comparing MetS patients with a control group, 6 for being reviews and 6 for not reporting results of control groups. Finally, a total of 14 articles met the inclusion criteria and were included in the final analysis.

### 3.2. Characteristics of eligible studies

MetS was defined when 3 or more of the following were satisfied: hypertriglyceridemia (≥150 mg/dL) or taking lipid-lowering drugs; elevated BP (≥85 mm Hg diastolic, ≥130 mm Hg systolic) or taking antihypertensive drugs; low high-density lipoprotein cholesterol (<50 mg/dL in women,<40 mg/dL in men); hyperglycemia (fasting glucose ≥100 mg/dL) or taking hypoglycemic agents drugs or insulin; and increased waist circumference (≥88 cm for women, ≥102 cm for men). Table 1 presents the characteristics of the 14 eligible studies, which include subjects' characteristics such as BMI, age, gender, and region; subjects' laboratory characteristics such as FPG, diastolic blood pressure, systolic blood pressure, HDL, and triglycerides; and circulating fetuin-A sample characteristics such as assay methods and storage temperatures. Altogether, these studies compared circulating fetuin-A concentrations between 4,551 MetS patients and 8,805 controls. The NOS scores of these studies varied between 5 and 8, with 8 studies graded as high quality and 6 as moderate quality.

**Table 1:** Characteristics of the studies included for the meta-analysis of fetuin-A and MetS.

Study	Material	Country	NOS	Male gender,n(%)	BMI	Mean Age	FPG	SBP	DBP	HDL	Triglycerides	Methods	Frozen
**(Can et al., 2016)** [34]	Serum	Turkey	8	18(42)	32.94±5.45	14.70±1.15	88.89±10.76	122.50±19.29	75.14±13.55	36.50±6.47	184.89±80.63	ELISA	-80°C
**(Huddam, Azak, Kocak, Bayraktar, & Sezer, 2013)** [35]	Serum	Turkey	7	8(32)	34.56±2.63	49.20±8.60	115.96±3.62	130.48±22.60	78.52±16.90	45.32±8.81	188.64±75.31	ELISA	-80°C
**(Nagwa Abdallah Ismail et al., 2012)** [36]	Serum	Egypt	6	6(41)	33.17±3.15	11.28±3.71	89.67±13.51	110.56±16.29	73.89±11.40	35.75±6.62	151.83±62.53	ELISA	NR
**(Nagwa Abdallah Ismail et al., 2012)** [36]	Serum	Egypt	6	14(40)	35.63±2.66	27.60±7.90	96.61±13.29	119.17±15.74	78.33±9.24	42.44±9.75	118.50±44.71	ELISA	NR
**(Ix et al., 2006)** [4]	Serum	USA	8	151(84)	27.00±5.00	69.00±12.00	81.00±29.00	86.00±15.00	58.00±18.00	33.50±1.40	99.30±23.50	ELISA	-70°C
**(Jialal et al., 2015)** [9]	Serum	USA	6	7(25)	36.0±6.0	51.00±11.00	100.00±10.00	132.00±12.00	83.00±9.00	41.00±9.00	132.00±103.00	ELISA	NR
**(Ju et al., 2017)** [23]	Serum	China	5	217(100)	28.58±3.16	48.57±12.25	95.83±18.4	137.84±18.25	86.47±13.19	27.7±9.52	173.03±12.08	ELISA	NR
**(Ju et al., 2017)** [23]	Serum	China	5	200(0)	27.84±3.22	48.28±11.74	95.36±17.7	134.88±19.29	85.16±12.93	33.25±5.49	112.35±11.9	ELISA	NR
**(Kaess et al., 2012)** [37]	Serum	USA	8	1705(47)	26.80±5.50	40.00±8.70	92.00±8.70	117.00±14.00	75.00±10.00	55.00±16.00	91.55±64.37	ELISA	-80°C
**(Kasabri et al., 2018)** [8]	Serum	Jordan	8	11(37)	31.20±7.20	46.00±3.78	86.00±7.98	135.00±13.00	84.00±23.00	38.00±5.00	199.00±26.00	ELISA	-80°C
**(Koch et al., 2013)** [38]	Serum	Germany	8	139(58)	25.90±2.33	65.00±5.80	84.50±7.70	140.00±120.00	80.00±7.80	33.00±7.00	93.00±56.00	ELISA	-80°C
**(Obuchi et al., 2014)** [39]	Plasma	Japan	5	43(26)	24.00±3.00	66.50±9.20	103.70±21.80	136.80±18.30	83.40±11.00	54.90±15.80	113.60±82.90	ELISA	NR
**(Roos et al., 2010)** [40]	Plasma	Germany	8	103(87)	27.00±3.40	60.70±6.90	105.70±27.30	120.90±16.10	72.90±9.00	36.88±9.10	140.00±110.00	ELISA	-80°C
**(Weghuber, Mangge, Hochbrugger, & Stulnig, 2014)** [41]	Plasma	Austria	5	36(66)	28.30±2.30	44.00±8.00	81.50±6.30	137.00±23.00	88.00±9.80	39.00±6.00	123.00±32.00	NR	NR
**(Weghuber et al., 2014)** [41]	Plasma	Austria	5	30(43)	29.20±3.50	13.00±2.90	71.20±5.50	85.00±13.00	61.00±11.00	31.00±5.00	91.00±16.00	NR	NR
**(Xu et al., 2011)** [42]	Serum	China	6	1367(56)	25.60±3.60	61.50±9.60	108.70±16.30	141.00±22.00	80.00±10.00	35.00±6.00	115.00±32.00	ELISA	NR
**(Zachariah et al., 2017)** [43]	Plasma	USA	8	378(50)	28.40±5.20	40.50±9.40	103.60±27.60	122.20±13.30	79.00±9.40	46.50±13.40	149.20±135.5	ELISA	-80°C

NOS, Newcastle-Ottawa Scale; BMI, Body Mass Index; ELISA, enzyme linked immunosorbent assay; NR, not report; FPG, fasting plasma glucose; DBP, diastolic blood pressure; SBP, systolic blood pressure; TG, Triglycerides; MetS, Metabolic syndrome; HDL,high-density lipoprotein. MetS was defined as the presence of 3 or more of the following: hypertriglyceridemia (≥150 mg/dL)or taking lipid-lowering drugs; elevated BP (≥85 mm Hg diastolic, ≥130 mm Hg systolic) or taking antihypertensive drugs; low high-density lipoprotein cholesterol (<50 mg/dL in women,<40 mg/dL in men); hyperglycemia (fasting glucose ≥100 mg/dL) or taking hypoglycemic agents drugs or insulin; and increased waist circumference (≥88 cm for women, ≥102 cm for men).

### 3.3. Overall comparison

**Fig 3:**
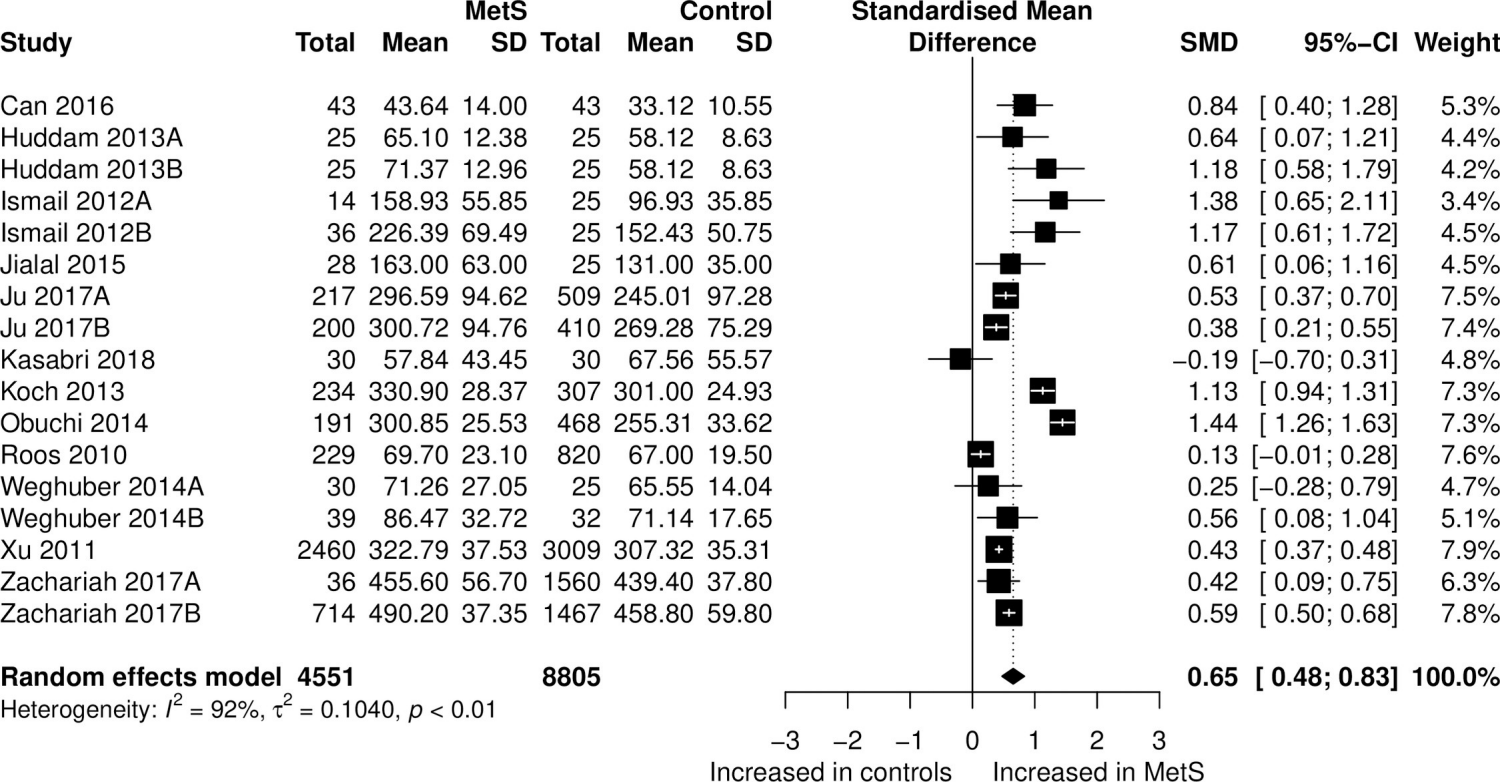
Forest plot of fetuin-A between MetS participants and controls. Study effect sizes of fetuin-A concentration differences between MetS and controls. Each data marker represents a study, and the size of the data marker is proportional to the total number of individuals in that study. The summary effect size for each fetuin-A concentration is denoted by a diamond. MetS, Metabolic syndrome; SMD, standardized mean difference.

Fig 3 presents the forest plot of the results of the SMDs in relation to meta-analysis using the random-effects model. Circulating fetuin-A concentrations were significantly higher in the MetS patients than in the controls (SMD = 0.65, 95% CI: 0.48 to 0.83, Z = 7.18, *p<*0.001). However, heterogeneity was considerable (*I*^*2*^ *=* 92.3%).

**Fig 4:**
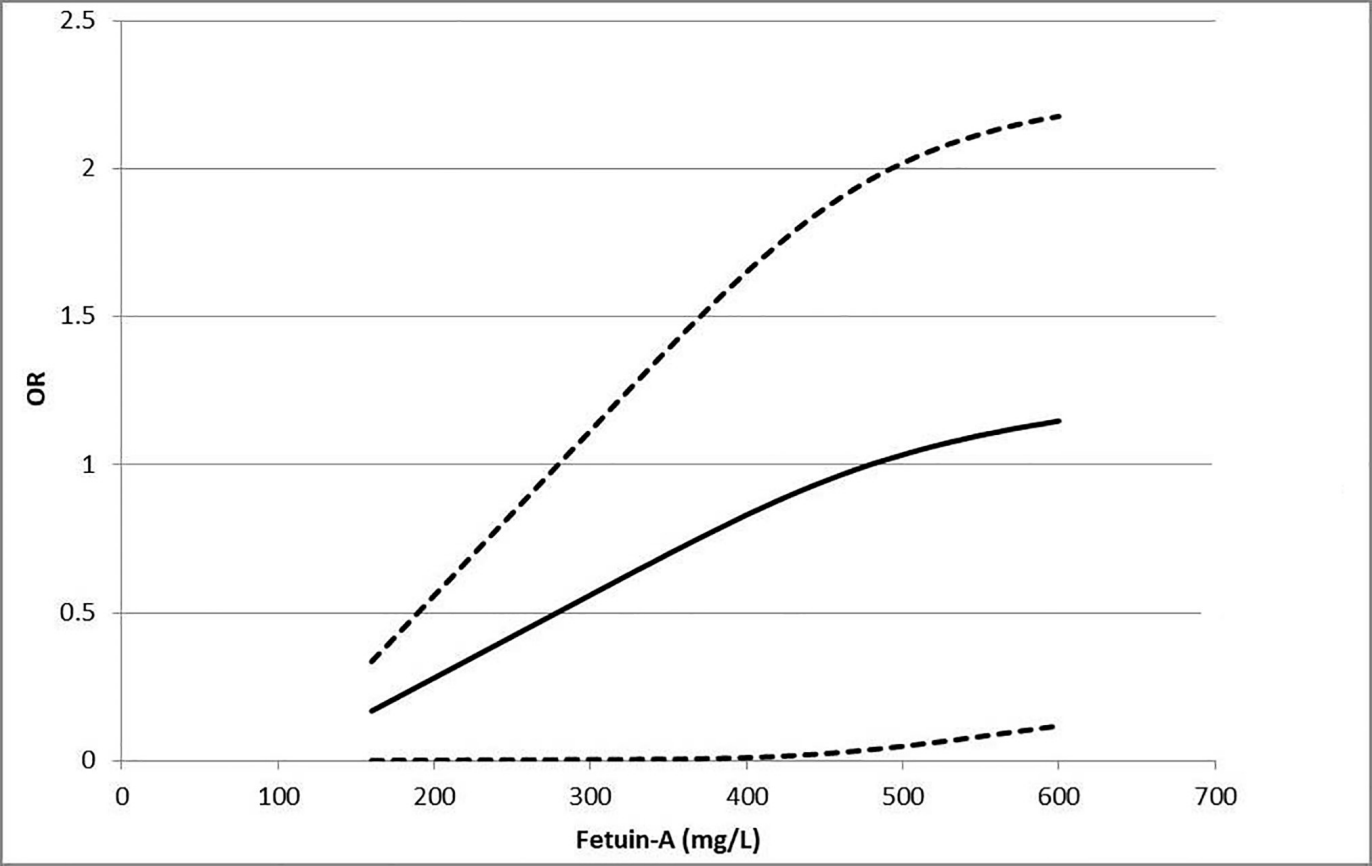
Non-linea dose–response relationship between the risk of MetS and fetuin-A concentrations. Odds ratio (OR;—) and the corresponding 95% CI (---) were summarized for thenon-linear dose–response relationship between fetuin-A concentrations with MetS risk. MetS, Metabolic syndrome; OR, odds ratio.

In the non-linear dose–response relationship, there was a slight trend towards an increment in the risk of MetS with an increment in the circulating fetuin-A concentration, although the increment was not statistically significant (p = 0.06). There was high heterogeneity (*I*^*2*^ = 71.7%) in the analysis of non-linear dose–response relationship between circulating fetuin-A concentration and the risk of MetS (Fig 4).The results of meta-regression analysis are shown in Table 2. The estimated amount of residual heterogeneity of meta-regression analysis (SE) was 0.1870, with *I*^*2*^ = 86.54%. Sample material, region, NOS, gender, BMI, age, FPG, systolic blood pressure (SBP), diastolic blood pressure (DBP), and triglycerides reporting were not significantly different. However, after introducing HDL into the meta-regression analysis model, results showed that sources of heterogeneity could be explained by HDL as the difference was significant. (*b =* −0.95, 95%CI: -1.83 to -0.07, *p =* 0.034).

**Table 2:** Meta-regression of the studies for the fetuin-A and MetS.

	Estimate	Standard error	Z value	p value	95% CI	
**Intrcpt**	1.8402	0.6119	3.0073	0.0026	0.6409	3.0396
**Material (Plasma = 0, Serum = 1)**	0.2277	0.4958	0.4593	0.6460	-0.7441	1.1995
**Region (Other = 0, Asia = 1)**	0.2645	0.3707	0.7137	0.4754	-0.4620	0.9910
**NOS (Other = 0, High = 1)**	-0.0641	0.3412	-0.1877	0.8511	-0.7328	0.6047
**Gender (Female = 0, Male = 1)**	0.3276	0.5227	0.6267	0.5308	-0.6969	1.3520
**BMI (<30 = 0, ≥30 = 1)**	-0.2076	0.7624	-0.2722	0.7854	-1.7019	1.2868
**Age (<18 = 0, ≥18 = 1)**	-1.0068	0.8061	-1.2489	0.2117	-2.5868	0.5732
**FPG (<100mg/dL = 0,≥100mg/dL = 1)**	-0.3789	0.4631	-0.8183	0.4132	-1.2865	0.5287
**SBP (<130mmHg = 0,≥130mmHg = 1)**	0.4204	0.4892	0.8594	0.3901	-0.5383	1.3791
**DBP (<85mmHg = 0,≥85mmHg = 1)**	-0.3779	0.6094	-0.6201	0.5352	-1.5723	0.8165
**HDL (≥40mg/dL = 0, <40mg/dL = 1)**	-0.9491	0.4482	-2.1177	**0.0342**	-1.8275	-0.0707
**TG (<150mg/dL = 0, ≥150mg/dL = 1)**	-0.2494	0.5934	-0.4203	0.6743	-1.4124	0.9136

NOS, Newcastle-Ottawa Scale; BMI, Body Mass Index; ELISA, enzyme linked immunosorbent assay; NR, not report; FPG, fasting plasma glucose; DBP, diastolic blood pressure; SBP, systolic blood pressure; TG, Triglycerides; MetS, Metabolic syndrome; HDL,high-density lipoprotein.

**Fig 5:**
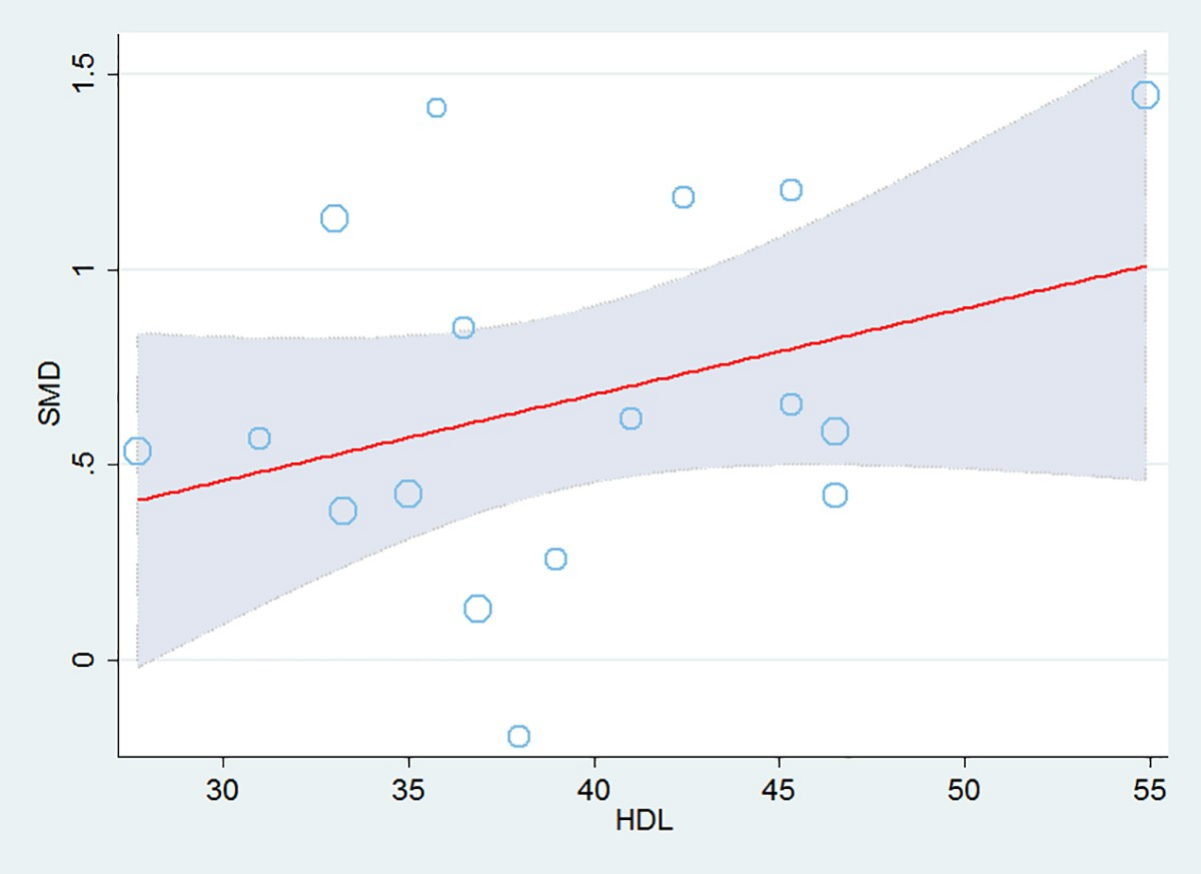
Dose-response relationships between HDL value index and SMD value outcomes based on data from each studies. Each data point overlaps into a circle. The size of the circle corresponds to the inverse variance weight of the SMD effect in the analysis.MetS, Metabolic syndrome; SMD, standardized mean difference.

The results of the analysis of dose-response relationship between HDL and SMD are shown in Fig 5. There was a positive dose-response linear relationship between HDL and SMD (*b* = 0.02, *p =* 0.014). Moreover, subgroup analyses were conducted to explore the impact of HDL, which showed that circulating fetuin-A concentrations were significantly higher in the MetS patients than in the controls for the two subgroups, high HDL (≥40mg/dL) concentrations subgroup (k = 7, SMD = 0.86, 95%CI: 0.48 to 1.25, Z = 4.39, *p<*0.001) and low HDL (<40mg/dL) concentrations subgroup (k = 10, SMD = 0.52, 95%CI: 0.32 to 0.72, Z = 5.01, *p<*0.001), but with high heterogeneity (*I*^*2*^ = 91.9% and 89.9%, respectively).

Table 3 presents the subgroup analyses for circulating fetuin-A concentrations between the MetS patients and the controls. Thus, most of the subgroup analysis results are consistent with the overall meta-analysis results, suggesting that these results are relatively stable. However, it is worth noting that age and DBP significantly explained the source of heterogeneity in relation to the outcomes. For example, circulating fetuin-A concentrations were significantly higher in the MetS patients younger than 18 years than in the control groups, but still with residual heterogeneity (I^*2*^ = 41.5%). Besides, the concentration of circulating fetuin-A was significantly higher in the MetS patients with DBP ≥ 85mmHg than in the control group, and most of the heterogeneity was explained (*I*^*2*^ = 7.7%).

**Table 3:** Subgroup analysis of the studies for the fetuin-A and MetS.

	Subgroup	SMD	95%-CI	p value	*I*^*2*^
**Material**	Plasma	0.5800	[0.1725; 0.9874]	0.64	95.9%
	Serum	0.6889	[0.4757; 0.9020]		87.6%
**Region**	Asia	0.7502	[0.4697; 1.0307]	0.31	93.3%
	Other	0.5377	[0.2437; 0.8316]		91.7%
**NOS**	High	0.5849	[0.2904; 0.8794]	0.51	91.7%
	Other	0.7240	[0.4398; 1.0081]		93.4%
**Gender**	Female	0.7943	[0.4121; 1.1765]	0.20	90.2%
	Male	0.5166	[0.3261; 0.7071]		92.5%
**BMI**	<30	0.6007	[0.3890; 0.8125]	0.43	95.1%
	≥30	0.7816	[0.3900; 1.1733]		71.2%
**Age**	<18	0.8547	[0.4540; 1.2554]	0.30	41.5%
	≥18	0.6187	[0.4259; 0.8115]		93.5%
**FPG**	<100mg/dL	0.6546	[0.3744; 0.9348]	1.00	86.3%
	≥100mg/dL	0.6557	[0.3974; 0.9140]		95.0%
**SBP**	<130mmHg	0.6311	[0.3644; 0.8978]	0.90	85.5%
	≥130mmHg	0.6561	[0.3762; 0.9360]		94.5%
**DBP**	<85mmHg	0.7159	[0.4988; 0.9330]	**0.04**	93.6%
	≥85mmHg	0.4510	[0.3288; 0.5731]		7.7%
**HDL**	<40mg/dL	0.5183	[0.3154; 0.7213]	0.12	89.9%
	≥40mg/dL	0.8627	[0.4776; 1.2478]		91.9%
**TG**	<150mg/dL	0.6471	[0.4175; 0.8766]	0.92	95.2%
	≥150mg/dL	0.6679	[0.3598; 0.9760]		68.6%

MetS, Metabolic syndrome; SMD, standardized mean difference; NOS, Newcastle-Ottawa Scale; BMI, Body Mass Index; ELISA, enzyme linked immunosorbent assay; NR, not report; FPG, fasting plasma glucose; DBP, diastolic blood pressure; SBP, systolic blood pressure; TG, Triglycerides; MetS, Metabolic syndrome; HDL,high-density lipoprotein.

Sensitivity analysis showed that the SMD and corresponding 95% CI changed little after each individual study was excluded sequentially, indicating that the overall results were relatively stable. The Egger funnel plot for circulating fetuin-A concentrations was symmetrical, and the Egger’s test did not reject the hypothesis that there was no publication bias (*t =* 1.21, *p =* 0.245).

Finally, all the results had moderate quality evidence. In this regard, methodological issues might have limited the overall quality of evidence. For example, the evidence of the difference in the circulating fetuin-A concentrations between the MetS patients and the controls was downgraded by one level because all plausible residual confounding would reduce the demonstrated effect; and the evidence of circulating fetuin-A concentrations, as a risk of Mets, was downgraded by one level because publication bias was suspected, and all plausible residual confounding would reduce the demonstrated effect, but with dose response gradient.

## 4. Discussion

The present meta-analysis revealed that there might be a relationship between circulating fetuin-A and MetS. That is, the circulating fetuin-A concentration in MetS patients was significantly higher than that in the control group. Therefore, subjects with high circulating fetuin-A concentration may have increased risk of developing MetS. Moreover, this study found that there was a slight trend towards an increment in the risk of MetS with an increment in the circulating fetuin-A concentration. Nevertheless, heterogeneity in the meta-regression, in relation to the foregoing results, was significantly high and substantially explained by HDL. Also, from the subsequent linear regression model test, a positive linear correlation was observed between HDL and SMD, indicating that HDL may play an important regulating role between circulating fetuin-A and MetS. This study also analyzed and summarized data from different age populations in order to see whether circulating fetuin-A concentrations could be affected by age differences [17].

The results suggested that the concentrations of circulating fetuin-A in the MetS patients was higher than that in the control group for all the age groups considered. However, meta-analysis with respect to the adult subgroup (18 years or older) was associated with significantly higher heterogeneity. Thus, it may be suggested that the activity of circulating fetuin-A, or the molecular mechanisms of circulating fetuin-A bioconversion were impaired in the adult MetS patients. Further investigations are necessary to understand the molecular mechanisms that account for the changes of circulating fetuin-A concentrations for different age groups among the MetS patients.

Furthermore, subgroup analysis showed that, for MetS patients with DBP≥85mmHg, the concentration of circulating fetuin-A was significantly higher than that of the control group, and this subgroup explained most of the heterogeneity. Considering that there were only three studies reporting DBP≥85mmHg, which represents limited available data thus far as regards MetS patients with DBP≥85mmHg, future research should expand the sample size to verify whether DBP plays a regulatory role in the MetS patients and circulating fetuin-A.

Many published reviews have suggested that circulating fetuin-A may play a role in the risk of type 2 diabetes mellitus, cardiovascular disease, and nonalcoholic fatty liver disease [18, 19]. For example, a meta-analysis on the association between type 2 diabetes mellitus and circulating fetuin-A showed that circulating fetuin-A concentrations in type 2 diabetes mellitus patients were significantly higher than in the controls [19]. This is in agreement with the findings of this study, suggesting that circulating fetuin-A may play a role in the disorder of glucose and lipid metabolism in humans with MetS. Although MetS share some features with type 2 diabetes mellitus or cardiovascular diseases, the role of circulating fetuin-A in MetS may be more complex [6].

For instance, free fatty acids and circulating fetuin-A play important roles in the interaction and metabolic processes between the liver and adipose tissues. According to some experiments, free fatty acids can increase the activity of nuclear factor κB (NF-κB) by restraining the AMP protein-kinase (AMPK), which in turn is a consequence of ERK 1/2 activation [20, 21]. Likewise, NF-κB up-regulates circulating fetuin-A gene expression and circulating fetuin-A mRNA expression, which may directly stimulate mechanistic target of rapamycin (mTOR) phosphorylation and sterol regulatory element binding protein-1c (SREBP-1C) expression [20, 22]. Moreover, available data indicated that up-regulation of lipogenic enzymes by SREBP-1C is beneficial to the accumulation of triacylglycerol. On the other hand, circulating fetuin-A inhibits the activity of insulin receptors and leads to insulin resistance. These effects are mediated through the PI3K and Akt signaling pathway [7].

Evidence from animal experiments has shown that elevated circulating fetuin-A concentrations could lead to impaired glucose control due to migration and activation of macrophage, impaired signaling of insulin receptors, triglyceride accumulation in hepatocytes, and dysfunction of adipocytes [23]. When the concentrations of glucose and free fatty acids in the blood of a MetS patient are increased, they can stimulate the binding of NF-κB to the circulating fetuin-A promoter, and then activate the expression of circulating fetuin-A mRNA to further promote circulating fetuin-A protein synthesis and secretion [20]. However, the inhibitory effect of adiponectin in this process has received attention recently. It is widely accepted that the adiponectin inhibits the expression of circulating fetuin-A induced by glycolipid disorder through AMPK pathway [24]. Therefore, the common hypoadiponectinemia in MetS patients may be another cause of increased circulating fetuin-A.

It has been proven from many clinical studies that circulating fetuin-A concentration in individuals with centripetal obesity is usually elevated. Given that centripetal obesity is closely related to insulin resistance and dyslipidemia, it is considered as a major sign of MetS [6]. The expansion of adipose tissue helps several pro-inflammatory mediators, such as macrophages and inflammatory cytokines (IL-6, TNF-α), to move into fatty tissue, where they interfere with the mechanisms by which adipose tissue responds to insulin action [25]. This leads to insulin resistance and insulin-mediated anti-lipolysis damage, which in turn lead to increased release of circulating fetuin-A from adipose tissue, excessive circulating fetuin-A uptake by the liver, and movement of circulating fetuin-A through the energy sensor AMPK, which is pivotal to directing hepatocytes to potentially deleterious pathways that lead to triglyceridemia [26]. Additionally, in a 1-year longitudinal follow-up study, gastric bypass surgery among morbidly obese patients reduced the concentration of circulating fetuin-A [27]. Another longitudinal study revealed that physical exercise intervention and weight loss practices were associated with significant reductions in fetoprotein levels [28].

The hypothesis of an association between MetS and circulating fetuin-A was supported not only by clinical and experimental studies, but also genetic epidemiological studies. For instance, it is widely accepted that the AHSG gene is located on chromosome 3q27, a region that has been identified as a MetS susceptibility locus. Thus, AHSG gene variant associated with lower circulating fetuin-A was more common in normal-weight men than in their overweight counterparts [5]. Therefore, the link between circulating fetuin-A and MetS appears to be at least partially mediated by genetics [29]. Other studies have shown that a single nucleotide polymorphism (SNP) of AHSG gene was associated with the prevalence of type 2 diabetes mellitus, and circulating fetuin-A concentration was negatively correlated with insulin secretion [30].

In this study, it was also shown that HDL played an important role in the relationship between MetS and circulating fetuin-A, which may be due to the fact that HDL is a possible component of dyslipidemia in MetS. However, it was further shown, through subgroup analysis, that there was significant heterogeneity among groups with different HDL concentrations and this was contrary to the results of the meta-regression analysis of HDL. The preceding contradiction may be caused by treating HDL differently in the subgroup and meta-regression analyses. For example, HDL was considered as a categorical variable in the subgroup analysis, whereas it was considered as a continuous variable in the meta-regression analysis. That is, taking HDL as a continuous variable might have increased the statistical efficiency of meta-regression analysis than considering it as a categorical variable in the subgroup analysis.

Thus, the results of subgroup analysis due to HDL should be seen as exploratory and interpreted cautiously. Nevertheless, recent studies also revealed that there was a correlation between HDL and circulating fetuin-A [31]. Moreover, experimental studies have shown that HDL can improve the lipid and glucose metabolism by activating the AMPK/SIRT1 signaling pathway [32]. Meanwhile, HDL can also promote glucose uptake in the adipocytes and glycogen synthesis through enhancing GLUT4 by mechanisms involving PI3K/Akt via AMPK signaling pathways [33]. In addition, AMPK pathway is the main pathway that inhibits circulating fetuin-A. Therefore, the common low levels of HDL in patients with MetS may be another reason for higher levels of circulating fetuin-A in this population.

### 4.1. Limitations

This meta-analysis and meta-regression are subject to several limitations. First, although there was a dose-response relationship between circulating fetuin-A and the risk of MetS, all the eligible studies analyzed were case-control studies, which could not make causality inference. In future, prospective cohort studies should be considered to explore the link between circulating fetuin-A and MetS. Second, majority of the eligible studies did not adjust for the possible confounding variables. Third, heterogeneity was high when synthesizing evidence on the relationship between the concentration of circulating fetuin-A and MetS. Therefore, future studies should consider investigating the association between the concentration of circulating fetuin-A and MetS in homogeneous populations using same methods. Last, only articles published in English were included. Therefore, this meta-analysis may be more prone to bias since some negative results are generally reported in regional journals in native languages.

## 5. Conclusions

Circulating fetuin-A concentration in the MetS patients was significantly higher than that in the control groups, and there was a slight trend towards an increment in the risk of MetS with an increment in the circulating fetuin-A concentration. Also, HDL may play a possible regulating role between circulating fetuin-A and MetS. Therefore, these results provide a rationale for evaluating circulating fetuin-A to see if it could affect the pathophysiological process of the risk of MetS. Specifically, this evaluation might lead to new perspectives in the early diagnosis, identification of novel biomarkers, and discovery of novel targets for pharmacological interventions.

## Supporting information

S1 File Search strategies. Details of search strategy.
(DOC)

S1 Table PRISMA checklist. (DOC)
