# Supplementary material for: Fetuin-A in Metabolic syndrome: A systematic review and meta-analysis
Source: PLoS One. 2020 Mar 5;15(3):e0229776. doi: 10.1371/journal.pone.0229776 (PMC7058339; doi:10.1371/journal.pone.0229776)
Supplement: S1 File — Details of search strategy. (DOC) [file pone.0229776.s001.doc]

Search strategies: details of search strategy. 3.2019

((Metabolic syndrome OR Metabolic Syndromes OR Syndrome, Metabolic OR Syndromes, Metabolic OR Metabolic Syndrome X OR Insulin Resistance Syndrome X OR Syndrome X, Metabolic OR Syndrome X, Insulin Resistance OR Metabolic X Syndrome OR Syndrome, Metabolic X OR X Syndrome, Metabolic OR Dysmetabolic Syndrome X OR Syndrome X, Dysmetabolic OR Reaven Syndrome X OR Syndrome X, Reaven OR Metabolic Cardiovascular Syndrome OR Cardiovascular Syndrome, Metabolic OR Cardiovascular Syndromes, Metabolic OR Syndrome, Metabolic Cardiovascular OR metabolism syndrome OR Insulin Resistance OR Syndrome X)) AND (alpha 2 HS Glycoprotein OR alpha(2) HS Glycoprotein OR alpha-2HS Glycoprotein OR alpha 2HS Glycoprotein OR alpha2HS Glycoprotein OR Fetuin-A OR Fetuin A OR Fetuin-B OR Fetuin B OR AHSG Protein OR alpha2 Heremans-Schmid Glycoprotein OR alpha-2-HS-Glycoprotein OR fetuins OR Fetuin)

376 of PubMed

(TOPIC: (((((((((((alpha 2 HS Glycoprotein OR alpha 2 HS Glycoprotein) OR alpha-2HS Glycoprotein) OR alpha 2HS Glycoprotein) OR alpha2ms Glycoprotein) OR Fetuin-A) OR Fetuin A) OR aisg Protein) OR alpha2 Heremans-Schmid Glycoprotein) OR alpha-2-HS-Glycoprotein) OR fetuin B) OR Fetuin-B) AND TOPIC: (((((((((((((((((((((Metabolic syndrome OR Metabolic Syndromes) OR Syndrome, Metabolic) OR Syndromes, Metabolic) OR Metabolic Syndrome X) OR Insulin Resistance Syndrome X) OR Syndrome X, Metabolic) OR Syndrome X, Insulin Resistance) OR Metabolic X Syndrome) OR Syndrome, Metabolic X) OR X Syndrome, Metabolic) OR Dysmetabolic Syndrome X) OR Syndrome X, Dysmetabolic) OR Reaven Syndrome X) OR Syndrome X, Reaven) OR Metabolic Cardiovascular Syndrome) OR Cardiovascular Syndrome, Metabolic) OR Cardiovascular Syndromes, Metabolic) OR Syndrome, Metabolic Cardiovascular) OR metabolism syndrome) OR Insulin Resistance) OR Syndrome X))

653 of Web of Science

('alpha 2 hs glycoprotein':ab,ti OR 'alpha(2) hs glycoprotein':ab,ti OR 'alpha-2hs glycoprotein':ab,ti OR 'alpha 2hs glycoprotein':ab,ti OR 'alpha2hs glycoprotein':ab,ti OR 'fetuin-a':ab,ti OR 'fetuin a':ab,ti OR 'ahsg protein':ab,ti OR 'alpha2 heremans-schmid glycoprotein':ab,ti OR 'alpha-2-hs-glycoprotein':ab,ti OR 'fetuin-b':ab,ti OR 'fetuin b':ab,ti) AND ('metabolic syndrome':ab,ti OR 'metabolic syndromes':ab,ti OR 'syndrome, metabolic':ab,ti OR 'syndromes, metabolic':ab,ti OR 'metabolic syndrome x':ab,ti OR 'insulin resistance syndrome x':ab,ti OR 'syndrome x, metabolic':ab,ti OR 'syndrome x, insulin resistance':ab,ti OR 'metabolic x syndrome':ab,ti OR 'syndrome, metabolic x':ab,ti OR 'x syndrome, metabolic':ab,ti OR 'dysmetabolic syndrome x':ab,ti OR 'syndrome x, dysmetabolic':ab,ti OR 'reaven syndrome x':ab,ti OR 'syndrome x, reaven':ab,ti OR 'metabolic cardiovascular syndrome':ab,ti OR 'cardiovascular syndrome, metabolic':ab,ti OR 'cardiovascular syndromes, metabolic':ab,ti OR 'syndrome, metabolic cardiovascular':ab,ti OR 'metabolism syndrome':ab,ti OR 'insulin resistance':ab,ti OR 'syndrome x':ab,ti)

336 of Embase

alpha 2HS Glycoprotein OR alpha2HS Glycoprotein OR Fetuin-A OR Fetuin A OR AHSG Protein OR fetuin-B OR Fetuin B in Title Abstract Keyword AND Metabolic syndrome OR Metabolic Syndromes OR Syndrome, Metabolic OR Syndromes, Metabolic OR Metabolic Syndrome X OR Insulin Resistance Syndrome X OR Syndrome X, Metabolic OR Syndrome X, Insulin Resistance OR Metabolic X Syndrome OR Syndrome, Metabolic X OR X Syndrome, Metabolic OR Dysmetabolic Syndrome X OR Syndrome X, Dysmetabolic OR Reaven Syndrome X OR Syndrome X, Reaven OR Metabolic Cardiovascular Syndrome OR Cardiovascular Syndrome, Metabolic OR Cardiovascular Syndromes, Metabolic OR Syndrome, Metabolic Cardiovascular OR metabolism syndrome OR Insulin Resistance OR Syndrome X in Title Abstract Keyword

25 of Cochrane
